# Adverse event assessment in a parenting programme: experiences from a multisite randomised controlled trial

**DOI:** 10.1186/s13063-024-08357-6

**Published:** 2024-08-17

**Authors:** Inga Frantz, Heather M. Foran, Jamie M. Lachman, Frances Gardner, Robert J. McMahon, Terje Ogden, Judy Hutchings, Madalina Ruxandra Costin, Ivo Kunovski, Marija Raleva, Janina Mueller, Nina Heinrichs

**Affiliations:** 1 Department of Clinical Psychology and Psychotherapy, Institute of Psychology, Universität Hamburg, Hamburg, Germany; 2 Institute of Psychology, University of Klagenfurt, Klagenfurt, Austria; 3 Department of Social Policy and Intervention, University of Oxford, Oxford, England UK; 4 Social and Public Health Sciences Unit, University of Glasgow, Glasgow, Scotland, UK; 5 Centre for Social Science Research, University of Cape Town, Cape Town, South Africa; 6 Simon Fraser University, Burnaby, Canada and BC Children’s Hospital, Vancouver, Canada; 7 Norwegian Center for Child Behavioral Development, University of Oslo, Oslo, Norway; 8 School of Psychology, Bangor University, Bangor, UK; 9 Department of Psychology, Babeș-Bolyai University, Cluj-Napoca, Romania; 10 Institute for Marriage, Family and Systemic Practice – ALTERNATIVA, Skopje, Macedonia; 11 Department of Psychology, Clinical Psychology, and Psychotherapy, University of Bremen, Bremen, Germany; 12 Department of Psychology, Bielefeld University, Bielefeld, Germany

**Keywords:** Harms, Adverse events, Negative effect, Parenting programme, Prevention

## Abstract

**Background:**

Clinicians and researchers should consider the expected benefits and potential harms of an intervention. Parenting programmes are a widely used evidence-based intervention for child behaviour problems. However, few data are available on potential negative effects. The aims of this paper were to increase systematic knowledge of adverse event (AE) assessment in parenting programmes and to provide an AE assessment tool.

**Methods:**

As part of the RISE project (prevention of child mental health problems in South-eastern Europe—adapt, optimise, test and extend parenting for lifelong health), we developed and tested an AE assessment procedure in three sequential studies for parents of children with child behaviour problems aged 2 to 9 years in North Macedonia, Republic of Moldova, and Romania. This paper reports on the development of the assessment tool in phase 1 (*N* = 140), phase 2 (*N* = 835), and the final experiences with using the optimised procedures in phase 3 (multisite randomised controlled trial, *N* = 823) in which AEs were assessed before, three times during intervention delivery, and at 1 year follow-up. At each time point, the participants completed a 12-item AE checklist. If moderate-to-severe problems of parent or child were reported, a structured follow-up interview was conducted.

**Results:**

The response rate on the AE assessment tool increased from 6% (phase 1) to 100% (phase 3) indicating improvement in collecting these data based on the experiences of each phase. Results of the RCT (phase 3) showed generally low (S)AE frequencies with the finally optimised procedure: During the intervention, no serious adverse events (SAE) were registered; at least one AE was reported by 10% (after the first session), 7% (after the third session), and 4% (after the last fifth session) of participants. None of the identified (S)AEs was causally related to the study or intervention. Cost–benefit considerations are needed to determine the best way to ensure participant safety in parenting programmes.

**Conclusion:**

The applied active AE assessment procedure provides a comprehensive AE assessment tool that can be used by others—with adaptations for the specific context, if needed. Based on our experiences, we outline recommendations for future studies.

**Trial registration:**

ClinicalTrials.gov, registration number phase 1: NCT03552250; phase 2: NCT03865485, phase 3: NCT04721730. Registered on 13 January 2021.

**Supplementary Information:**

The online version contains supplementary material available at 10.1186/s13063-024-08357-6.

Since the 1990s, there have been increasing calls to systematically assess the benefits of psychological interventions. A rich body of high-quality psychological trials have been conducted over the last 30 years. Today, guidelines for the conduct and reporting of clinical trials (e.g. CONSORT) [1, 2] assist in reflecting on large numbers of rigorous studies, as well as meta-analyses, demonstrating the beneficial effects of psychological treatment across a variety of mental disorders, (sub)clinical problems, and settings. However, psychological treatments should not only be effective but also safe, and clinical treatment guidelines need to ensure that evidence of both benefits and potential harms of interventions is collected [3, 4]. Besides the treatment itself, also non-intervention trial procedures, such as repeated assessments, randomisation, or wait-lists can have unwanted effects [1]. There is some evidence for harms (i.e. in the frequency of adverse events, AE) in indicated prevention as well as psychotherapeutic trials. For example, there are studies in which youth at risk for anti-social behaviour showed higher levels of delinquency, alcohol, and drug use and criminal behaviour after a group-based skills training [5, 6]. Linden (2013) estimated that overall, 3 to 15% of psychotherapy patients experience adverse reactions, such as the emergence of new symptoms, changes in family relations, and sick leave during or after therapy [7]. Other authors [8] report even higher numbers. Many different terms are used to describe potential negative or unwanted intervention effects. Where possible, we apply the terms suggested by the CONSORT extension for reporting harms in trials [1, 2] (Table 1).

**Table 1:** Definitions of harms and applied adaptation for parenting programmes

Term	Definition	Adaptation for Parenting Programme Intervention Type
Harms	Overall term for all negative consequences that a treatment can have [1]	All negative reactions (see below) as well as burdens
Burden	Treatment-related activities	May include homework or skill practice and time involvement (e.g. number of sessions). Assessment-related burden may include time for assessment
Adverse events (AE, or unwanted events) [6]	Negative events that occur in parallel to an intervention—independent of the causality [1]. AE could include new physical and psychological symptoms, as well as problems in social life or work performance AE can be expected [1] or unexpected	Any significant mental, medical, psychosocial, or cognitive health problem of the participating parent or the child that has newly occurred or that has remarkably worsened (i.e. new occurring symptom or worsening of a pre-existing problem) compared to baseline. This may include physical problems (e.g. broken leg), emotional problems (e.g. higher levels of anxiety), behavioural problems (e.g. aggressive behaviour, alcohol misuse); difficulties in daily life that interfere with personal relationships or ability to fulfil daily tasks (e.g. cannot go to work) Expected AE: an initial increase of child mental health problems when new parenting strategies are implemented for the first time. For example, when participating in a parenting programme, the parent might shift his/her attention towards the behaviour problems of the child. This can lead to increased levels of parent-reported child behaviour problems. Also, it is possible that participants may experience moments of sadness when discussing difficult situations, like violence in the family or the death of a family member. Also, parents might argue about the new parenting [8]. These negative effects are expected to only last for a short period of time Unexpected AE: e.g. parent reports new back pains during the parent training
Serious adverse events (SAE)	Very severe forms of negative events (e.g. life threatening) that may include hospitalisation, suicidal ideation or attempt, and violence towards self or others [4]	AE associated with severe consequences for the participating parent or child. This could be any life-threatening event or events that may lead to hospitalisation (for at least one night) or death
Adverse reactions, negative effects, or treatment-emergent reactions [1, 7]	AE that are caused by or causally related to the intervention	AE is causally related to intervention (e.g. escalation of child behaviour problems when new parenting strategies are implemented for the first time; new interparental conflicts about parenting strategies/interparental violence due to parenting issues

## Relevance of adverse event (AE) assessment in parenting programmes

Parenting programmes come in various formats (e.g. groups vs. individual) with numerous theoretical backgrounds, as universal, selective, or indicated prevention programmes as well as additional intervention support in the context of family mental health treatment services. Parenting programmes are frequently used in the context of child mental health problems, for example child anxiety, depression, attention-deficit hyperactivity disorder (ADHD), autism, and child conduct problems (e.g. oppositional defiant disorder, ODD, conduct disorder, CD) [9]. These programmes can often be used with any caregiver of children (e.g. grandparents, foster parents) who is spending a significant proportion of time with the child and is sharing everyday life, at least for the majority of the week. We refer to these caregivers who are usually attachment figures by the term ‘parents’. This is inclusive and also reflects that the majority of children are indeed living with at least one (biological) parent. In this paper, we studied a parenting programme for parents of children with behaviour problems.

Whilst the collection and analysis of AEs is incorporated as standard procedure in most randomised controlled trials (RCTs) within health research, reporting of potential negative events is much less common in mental health trials and parenting research specifically. This may be grounded in the assumption that psychological interventions—in contrast to medical treatment—are unlikely to cause harm. However, it is equally important to assess AEs in this type of study because (1) every intervention and study are associated with at least some burden (e.g. burden of time for the intervention and/or assessment) and (2) the harms and intervention-related burdens can outweigh the benefits. Even in preventive interventions with low-risk families, safety reporting is crucial as the effectiveness of interventions might be smaller and therefore skew the risk–benefit evaluation towards a more conservative approach. This reinforces the significance of also collecting AE data in low-risk samples [1]. As a result, Bonell and colleagues [10] recommend an assessment of harms across all levels of public health interventions to establish their existence and prevent adverse effects across different levels of intervention intensity.

## Current AE assessment and reporting practice in parenting programmes

Given the relevance of harm assessment, recent guidelines for clinical trials (including all psychological and medical studies) require the assessment and reporting of AEs and negative reactions. For example, the Declaration of Helsinki and the American Psychological Association’s (APA) Journal Article Reporting Standards (JARS) [11] require investigators to assess burdens and risks during clinical trials. Moreover, an extension of the CONSORT statement outlined detailed standards on how to report potentially harmful AEs in RCTs [1].

A review of reviews by Barlow and Coren [12] concluded that there are no data available regarding potential AEs in parenting interventions. Another review of evaluation studies for parent trainings in children with ADHD [13] also noted a lack of AE reporting [14]. More recently, a small number of RCTs on parenting interventions studies have comprehensively reported on AEs. For example, Bearss et al. [15] compared a parent training and a parent education intervention for children with autism spectrum disorder. They assessed AEs using an active surveillance method (form) at each of the 12 sessions. In addition to 533 moderate-to-severe AEs, they reported three serious adverse events (SAEs, in 180 families, 2% across conditions). Another study, a cluster RCT, of a home-visiting prevention programme in a low-resource setting, reported severe risk of harm in 2% of the sample (e.g. suicidality of caregiver) [16]. Specifically, in each of the two groups (intervention and control group), 12 cases were identified between baseline and post-assessment.

Clinical research on psychotherapy suggests that the risk of AE is higher in samples with more severe problems [8]. Based on existing evidence from studies on parenting programmes, the risk seems rather low with SAE frequencies overlapping with the lower border of the range of adverse reactions in psychotherapy trials [7]. One explanation for this relatively low number of AE reports in psychological trials in general—and in parenting programmes specifically—might be because psychological interventions, especially preventive ones, are often considered to be safe. Also, there is a ‘lack of clear guidelines on how to define, categorize, identify and report harms within this research field’ (of psychological interventions, [17] p. 2). Reported frequencies of AEs may be highly dependent on how the data are collected (i.e. how participants are instructed to report on these events; who is collecting the data, e.g. therapist or facilitator vs. member of the research team). This can be via an open-ended question at the end of the intervention or repeated throughout the intervention, addressing each person individually or offering separate follow-up contact if a therapist perceives a participant worsening. Assessments of AE can also involve use of a checklist that routinely reminds participants to evaluate all areas in which an AE could occur (active assessment).

In addition to the method of collecting these experiences, differences in AE definitions might also generate differences in SAE frequencies across studies. For instance, ‘symptom worsening’ is often an example of an AE in studies focusing on mental health. In a parenting intervention, this may relate to the child, the primary caregiver, both or potentially, if viewed from a systemic standpoint, other core family members. Furthermore, the most proximal variable targeted in parenting programmes is parenting behaviour. Worsening of such behaviour is certainly of primary interest during a parenting intervention but not necessarily during other types of interventions. Unfortunately, reports on AE assessment methods mostly lack sufficient detail to allow comparison or replication of findings across studies.

Another challenge in AE assessment is how to disentangle adverse reactions from short-lived, expected side effects in parenting programmes, events that may arise during a correctly conducted intervention (e.g. escalating child behaviour problems when a parent implements time out for the first time) and more negative effects of the intervention (e.g. permanent increase of child behaviour problems due to ineffective programme or increased risk of intimate partner violence experience due to partner conflicts about parenting styles) [7, 18]. These effects may only be differentiated based on a priori assumptions about the mechanisms of change of such interventions. In addition, like other intervention types, it can sometimes be difficult to decide if an occurrence is positive or negative in a parenting intervention (e.g. divorce from partner) [7]. Further, we do not know when the adverse effects are most likely to occur (e.g. during or after the intervention), which are the most common ones, and how these can be reliably assessed.

In sum, collecting and analysing data on AEs in parenting interventions is challenging: Parenting programmes implemented as (targeted) prevention might not be evaluated as a ‘clinical trial’ and therefore researchers (and editors) might conclude that AE assessment is not necessary or that guidelines for clinical studies do not apply. In contrast to pharmacological interventions and individual psychotherapies, parenting programmes include not only the index person (i.e. the child) but also the caregiver. As these programmes are usually aiming to have positive effects on both children and parents, it is important to assess potential harms in both groups. Furthermore, to our knowledge, there are no standard instruments available for AE assessment in parenting programmes. Most of the available AE tools in psychotherapy research [8] are quite lengthy (i.e. 16–147 items) which might make it difficult to repeatedly assess in brief interventions (e.g. 10 sessions or less). Finally, the available AE tools focus on the potential harms and burdens of an index patient but not of (other) family members [19]**.**

To conclude, we need to increase systematic knowledge about potential harms in psychological interventions in general and parenting programmes specifically. As psychological interventions differ widely in their expected effect—depending on symptom severity (prevention vs. intervention), targeted problems (e.g. individual depressive symptoms vs. substance abuse vs. marital conflicts), targeted persons (only index person vs. other persons included), and type of intervention (e.g. in vivo exposure therapy of index person vs. family intervention)—it is recommended that each research domain outlines the exact definitions, assessment methods, and reporting standards for AEs in each specific field [20, 21]. In the field of parenting interventions, it is important to develop standard instruments that systematically assess AE in an economic, valid, and feasible way for, usually, short-term interventions. It might be more promising to use active (e.g. with a questionnaire) instead of passive (e.g. only note any spontaneous reports by the caregivers) surveillance because of the lower risk of missing any potential negative events.

## Aims of this paper

This paper aims to share experiences of developing and testing an AE assessment procedure used in an evaluation of a parent intervention with two goals: (1) to make a stepwise approach to the systematic assessment of AEs in parenting intervention studies that are economic, feasible, and inclusive (i.e. allow caregivers with language difficulties/low literacy to self-report potential AEs by using symbols) and (2) to share the AE tool including results from the final optimised version of the assessment method used in a multisite RCT that evaluated a parenting intervention for parents of children with increased levels of behaviour problems. For reporting, we apply CONSORT harms extension [1, 2] and, where appropriate, make suggestions for adaptations in the field of parent interventions (Table 1). We assumed that we would find (1) higher AE response rates with the optimised AE procedures in phase 3 compared to the prior study phases (i.e. less missing AE data; definition response rate: participants with valid AE data out of all families that participated per assessment point) and (2) similarly low rates of SAEs as reported previously (around 2 to 5%) [15, 16]. We expected some association of (S)AE occurrence and mental health symptoms in the family (i.e. families with higher levels of mental health problems were at higher risk for experiencing (S)AEs. We were also interested in exploring how frequent different categories of problems (emotional, physical, and social problems as well as other problems in daily life)—as newly occurring or worsening during the study—were reported by parents (independent of whether the reported problem then met the criteria for an AE or SAE). Potential expected (temporary) negative effects of parenting programmes were a priori defined as (short-term) increases of aggressive child behaviour (e.g. initial increase of oppositional behaviour when new parenting strategies are implemented for the first time), emotional distress in parents (e.g. because they do not feel yet comfortable with the new parenting strategies), and interparental conflicts (e.g. because parents disagree on the new parenting strategies). Finally, we were interested in identifying adverse reactions (i.e. parenting intervention study-emergent reactions) and side effects (unintended effects caused by the intervention or study).

## General method

### Overview: the RISE project

In the RISE project (prevention of child mental health problems in south-eastern Europe—adapt, optimise, test, and extend parenting for lifelong health), we developed, tested, and evaluated a parenting programme for the specific needs of families in south-eastern Europe across three consecutive phases (applying the multiphase optimisation strategy, MOST) [22]. In each phase, we implemented the Parenting for Lifelong Health Programme for Young Children (PLH-YC) [23, 24], a group-based programme based on social learning theory for parents of children aged 2 to 9 years with elevated levels of child behaviour problems. Implementation sites were community centres, health clinics, and schools in the Republic of Moldova, North Macedonia, and Romania.

#### Participants

Inclusion criteria [23, 25, 26] were parents or primary caregivers who (1) were ≥ 18 years of age and with children aged 2 to 9 years, (2) reported at least subclinical levels of child behaviour problems (scores ≥ 10 on the oppositional defiant disorder subscale, ODD; on the Child and Adolescent Behavior Inventory, CABI) [27], (3) spent at least four nights a week with the child in the same household, (4) agreed to being randomised to one of the conditions (phases 2 and 3 only), (5) consented to participate in the full study, and (6) had adequate language skills to participate in the intervention, either in the primary language of the group or with additional language support provided. (1) We excluded primary caregivers whose children were removed from their custody.

#### AE assessment procedures

(1) Potential AEs were assessed using parents’ self-report. In phase 1, parents answered to an open-ended question. In phases 2 and 3, parents completed an AE checklist (see below). (2) Parents’ self-report was adjudicated in a follow-up interview to decide whether the criteria for an AE or SAE (Table 1) were met.

#### AE reporting procedures

In case of any reported (S)AEs, research personnel sought more details about what happened and informed the local principal investigator (PI) within 24 h. The PI adjudicated the report (e.g. criteria for AE or SAE met, increased risk for participants, AE/SAE anticipated or unanticipated), decided on further actions, and reported the (S)AE to the project coordinator (NH, IF). The research personnel and PI also evaluated whether the (S)AE was causally related to the study (meaning an unwanted negative effect vs. problem with other cause; this was independent of whether the problem was anticipated or unanticipated). When local legal criteria for child maltreatment were met, the PI also reported to local child protection services. In cases of an SAE, the PI informed the local ethical institutional review board (IRB) and the project coordinator informed the independent data safety and monitoring board (DSMB, consisting of two experts in the field of parenting interventions, TO and RJM), with assistance of the PI HMF (who managed all IRB submissions for the overall project at the University of Klagenfurt IRB)—the central IRB in Klagenfurt. Based on the information given, the DSMB members (1) decided whether the study could proceed without evidence or risk of unanticipated harms to participants and how the study protocol or consent needed to be changed and (2) stopped the trial if it was deemed that risks of the study outweighed benefits.

## Phase 1

### Methods

Phase 1 included a pre-post feasibility study (*N* = 140) [25, 28] to test the assessment, implementation, and intervention procedures in the three countries (data collection: pre: April to June 2018; post: September to December 2018). The PLH-YC programme was translated and adapted to the specific context. The 12-session version was delivered in North Macedonia and Republic of Moldova, whilst in Romania, a condensed 6-session version was used (to ensure that the programme was completed before the summer holidays) [25, 28].

#### AE instrument

We used an open-ended question for AE assessment (informed by the procedures of the STRONG STAR and GROW&TREAT studies) [29, 30], such as ‘How are you doing—have you or your child had any problems since the last contact with anyone from the project team?’ During the intervention, the programme facilitators asked the participants at each contact (individual and group sessions) about potential AEs since the last session. At post-assessment, research assistants administered the AE question as part of the outcome assessment interview. If a potential AE was reported, local research staff assessed further details (e.g. severity, actions taken, relatedness to study; see ESM 1) and decided if the criteria for an AE or SAE were met (AE/SAE criteria phase 1, see ESM 1).

#### Participants

From 140 families enrolled, 94 completed post-assessment. Mean age of the index child ranged from *M* = 5.5 (SD = 2.1, Romania) to *M* = 6.3 (SD = 2.1, Republic of Moldova; North Macedonia *M* = 5.7, SD = 1.8); parents’ age ranged between 34 (SD = 7.5; Republic of Moldova) and 37 years (SD = 4.3; North Macedonia; Romania *M* = 34.6, *SD* = 9.8). Regarding the education level, 75% of the parents from Romania had no college degree (North Macedonia 36%, Republic of Moldova 35%); and 47% could not/only read with difficulties (North Macedonia 12%, Republic of Moldova 9%) [28].

## Results and discussion

In phase 1, we received AE reports from a small number of families and assessment points (*n* = 7 during the intervention out of 119 allocated to group, 6%, *n* = 10 out of 94 families assessed at post-intervention; AE post-intervention response rate 11%) [28]. AEs included medical problems (e.g. high blood pressure), interpersonal problems (e.g. parent slapped child), and emotional problems (e.g. worries about relative with severe illness, marital conflicts). Across the 2 assessment points, 5 SAEs were reported (3 recorded during the intervention, 2 at post-assessment; a total of 5 out of 140 participants: 4%) [28]. SAEs were hospitalisation because of medical problems (e.g. cardiovascular problems) or interpersonal problems (injury due to physical aggression by peers). None of the SAEs was an adverse reaction caused by the intervention or study. Based on other reports from participants (e.g. in questionnaires or in post-intervention focus groups), we additionally realised that it was likely that more events happened that could have been reported. Feedback from the research coordinators suggested that the procedures (setting out when and how to complete the AE forms and forward them to the PI and afterwards to the coordinator) were not followed due to misunderstandings, time constraints, or subjective evaluations of the insignificance of the reports (lack of sensitivity) by research staff.

## Phase 2

### Methods

Phase 2 comprised a factorial cluster randomised trial to test different PLH-YC programme and implementation components [23], (Foran HM, Lachman JM, Zhao X, et al: A cluster randomized factorial trial of the Parenting for Lifelong Health program for Young Children: results from the optimization phase of a Multiphasic Optimization Strategy, submitted). Pre-assessments began in March 2019 and were completed by mid-April 2019. Programme delivery was face-to-face in spring 2019. Post-test assessments were conducted from September to December 2019 with follow-up assessments took place from January to April 2020 in North Macedonia and Romania and from March to May 2020 in the Republic of Moldova. Because of the restrictions due to the pandemic, we switched assessment mode from in-person interviews to phone calls for follow-up assessments, if needed.

#### AE instrument

Based on experiences during phase 1, we revised the AE assessment procedures for phase 2 with the following specifications: we used a standardised AE checklist to make sure that we received data from all participants at all assessment points. To facilitate the AE assessment during the group sessions, we used a self-report tool for parents. We applied symbols and minimised language to account for low literacy levels. For the categories on the AE checklist (e.g. headache, accident, feeling sad, or depressed), we used examples reported from phase 1. These included five physical/medical problems (e.g. injury, headaches), four behaviour problems (e.g. aggressive behaviours), four emotional problems (e.g. depression; answer format: happened to parent/child no/yes, if yes severity rated from 1 = mild to 4 = severe); and five problems in daily life (e.g. emergency room visit). In order to assess any negative effects that occurred after programme completion, we also assessed AEs at a 6-month follow-up.

#### Participants

At baseline, *N* = 835 participants were assessed. From these families, *n* = 735 were enrolled in the programme and participated in at least one programme session; *n* = 661 participated at post-assessment, and *n* = 582 participants at 6-month follow-up. In the included families (*N* = 835), parents’ ages ranged between *M* = 36.0 (*SD* = 6.4) and *M* = 36.5 (*SD* = 6.6) years across conditions; parents’ gender was mostly female (95-97%). Sixty to sixty-four percent of participating parents had a college/university degree (35-39% some schooling, 0-1% no schooling). Regarding the index child, age ranged between *M* = 5.5 (*SD* = 1.9) and *M* = 5.8 (*SD* = 2.0) years across conditions with slightly more boys than girls (58-63% boys).

## Results and discussion

After piloting these procedures, we received numerous notifications that needed follow-up and ended up being evaluated as non-significant (overly sensitive; e.g. the parent had migraine attacks for years, hence, the problem was not new or worse than usual). To address this, we changed the procedure and introduced a severity threshold for each event and if this was met (severity of 3 or 4 on checklist, or a problem in daily life), an adjudication interview was conducted (ratings conducted for each reported event separately).

In phase 2, the AE response rates were higher compared to phase 1: the overall coordinator (PI: last author) received a total of 606 completed AE checklists from the three country sites. However, most of these were from post- and follow-up-assessment (post-assessment: 451 out of 661 completed assessments; AE response rate 68%; follow-up assessment 152 out of 582 completed assessments, AE response rate 26%). The response rate on the AE assessment during the intervention was low (interim assessment: one from the 735 families enrolled in the programme, < 1%; pre-programme consultations: two from *N* = 735 families enrolled). Based on feedback from the programme coordinators, it was not feasible for programme facilitators to complete the AE checklist with parents during the group sessions because of time pressure and other competing tasks whilst delivering parenting sessions.

Research staff conducted AE follow-up interviews with 82 families (2 during pre-programme consultations, 1 during intervention, 50 during post-assessment, 29 during follow-up period). During the pre-programme consultations, two AEs were coded in two families (both happened to the child, one expected, one unexpected, both unrelated to the study). During the intervention, one SAE occurred: one child died unexpectedly (unrelated to the study). At post-assessment, 27 AEs and 4 SAEs were identified during the follow-up assessments (all unrelated to the study; in 19 cases the criteria for SAE/AE were not met after the adjudication interview; e.g. because the problem did not newly or occur or got worse). During the follow-up assessment, 24 AEs and 3 SAEs were identified (all unrelated to the study, in two cases criteria for AE not met).

## Phase 3

### Methods

Families were randomly assigned to the intervention (five sessions PLH-YC parenting programme group) or the control group (one session lecture on parenting; https://rise-plh.eu/about-lecture-intervention-2/). The intervention trial was conducted during the COVID-19 pandemic and was shifted to mostly remote assessment and online intervention delivery via Zoom [26]. Families were recruited from December 2020 until February 2021.

Across the three countries, a total of 823 families were assessed and randomly allocated to the PLH-YC (*n* = 415) or lecture (*n* = 408) condition. Participating parents were mainly female (97% in both groups), about 35 years of age (PLH-YC group: *M* = 35.4, SD = 5.1; lecture group: *M* = 35.6, SD = 5.3). Child mean age was 5 years (PLH-YC group: *M* = 5.2, SD = 2.1; lecture group: *M* = 5.1, SD = 2.0). Baseline levels of the aggressive behaviour subscale of the Child Behavior Checklist (CBCL) [31] were approximately half a standard deviation above the mean (*t-*scores*:* PLH-YC group: *M* = 55.5, *SD* = 12.0; lecture group *M* = 55.1, SD = 11.0). Regarding clinical levels of child behaviour problems, 8.5% in the PLH-YC group met diagnostic criteria for ODD or conduct disorder (CD assessed with the MINI-KID-P) [32], compared to 7.4% in the lecture group. There were no baseline differences regarding any of the assessed outcomes and demographic data between the two groups (34).

#### AE assessment

Based on the experience in phase 2 and feedback from research staff at implementation sites, we further optimised the AE assessment procedures for phase 3. This included shortening the AE checklist (e.g. items were merged, for example back pain and headache were merged to one item assessing pain) and an adaptation of the adjudication form (e.g. specification of criteria for (S)AE; adding outcome). We also changed the assessment procedures to administer the AE checklist in individual assessments during the intervention (phone calls independent of the parenting programme sessions). This allowed for greater differentiation between the implementation and research components of the study, thus increasing the likelihood of reporting AE during intervention delivery. Also, this allowed the use of identical procedures in the intervention (five sessions) and control group (one session).

Following the recommendations by Linden (2013) [7], we considered all AE that occurred in parallel (with a time connection) with the intervention study using a standardised checklist, because all occurring negative events could potentially be caused by the intervention or study. In a second step, more details were gathered in an adjudication interview. This included an evaluation of whether or not the AE was related to the intervention or study and hence classified as an adverse reaction or not (Table 1).

##### AE checklist

All parents were asked to complete the self-report checklist five times: at pre-assessment, three times during the intervention, and at a 12-month follow-up. The checklist consisted of 12 items assessing physical problems (three items: accident, injury, pain), behavioural problems (three items: aggressive/violent behaviours, sleep disturbances, pain), emotional problems (one item: emotionally distressed), and other significant problems in daily life (five items: difficulties with personal relationships, unplanned hospitalisation, emergency room visit, death of a loved one, any other problems).

For each item, the parent reported whether this had happened to him/herself or the child (yes/no) since the last contact with the assessor or facilitator (usually 2 weeks; at pre-assessment we asked: ‘in the last four weeks’ before this baseline assessment). During the intervention and at the follow-up assessment (conducted about 6 months after the AE assessment following the fifth (final) session/4 weeks after the lecture) we asked ‘since the last interview’ so as to not miss any (S)AEs that happened during/after the completion of the programme and might be causally related to the study participation. If a parent reported a problem on the checklist, he/she then rated its severity (from 1 = mild to 4 = severe, answer-format for problems in daily life: yes/no; full checklist, ESM 2).

##### Adjudication interview

Based on the experiences of the previous phases of the RISE project where parents also reported mild events on the checklist that did not necessarily meet the AE criterion of a significant deterioration (noteworthy worsening of an existing problem or a significant new problem), we only followed up reports of behavioural, physical, and emotional problems with moderate or severe severity (3 or 4 on the checklist), or a significant problem in daily life. If this threshold was met, a semi-structured follow-up interview (ESM 2) was conducted to find out more about what had happened and to decide whether or not the definition of an (S)AE was met.

During the interview, parents provided a detailed description of the event and the research personnel recorded whether any actions were taken (e.g. offer additional 1:1 counselling), the outcome of this action (e.g. 1 = recovery to 5 = death, 6 = unknown), and to whom the AE happened (child/parent), whether the AE was (un-)expected and its severity (from 1 = mild to 4 = severe). Also, the causal relationship to the study was rated based on the detailed parent report (1 = not related to the study, 5 = definitely related to the study; for complete follow-up form, see ESM 2). If parents reported more than one problem (e.g. worsening of aggressive child behaviour, parents’ newly occurring emotional distress), the follow-up form was completed for each potential AE separately.

#### AE procedures

At pre- and follow-up assessment, the AE checklist was administered as part of the main outcome measure assessments. These assessment meetings were conducted in-person or over the phone/or video (depending on local COVID-19 restrictions) by local data assessors [26]. During the intervention, three phone calls were conducted to assess child and parent behaviour and the AE checklist was administered, after the first PLH-YC session (intervention group) or the lecture (control group); the third PLH-YC session (or for the control group 2 weeks after the lecture), and the last PLH-YC session (fifth session, or for the control group 4 weeks after the lecture). The data assessor entered the parents’ responses to the checklist into a tablet (via Open Data Kit software). The follow-up form was completed by the data assessor via paper–pencil.

#### Additional validation measures

Child mental health problems were assessed with the CBCL (total score; CBCL 1½–5 version: 100 items, CBCL 6–18 version: 113 items) [31]. Parents reported on a 3-point Likert scale; raw scores were transformed to age-adjusted standardised *t-*scores (based on multisocieties including Romania). Higher CBCL scores represented more mental health problems. Parent mental health problems were assessed using the DASS-21 [33]. Sum score ranges from 0 to 63 with higher scores indicating more depressive, anxiety, or stress symptoms. Social support was assessed with the emotional support subscale of the Medical Outcomes Study (MOS) Social Support Survey (eight items, mean score transformed to a 0 to 100 scale with higher scores indicating more perceived support) (Heinrichs N, Lachman, JM, Waller F, Müller M, Frantz I, Raleva M, et al: Multi-country randomised controlled trial of a systematically optimised, remotely-delivered five-session parenting intervention with parents of children ages 2–9 years with conduct problems in Eastern Europe, submitted). For full description of measures, see Taut (2021) and Heinrichs (2024) [26, 34].

## Results

### Validation of AE procedures

#### Response rates

In phase 3, the optimised AE procedures yielded higher response rates compared to phases 1 and 2 (Table 3). Especially data collected during programme delivery indicated that the AE response rate increased from 6% (phase 1) to almost 100% (phase 3; 648 AE data available out of 649 participants after the first session). At follow-up assessment, the AE response rate was 99% in phase 3 (527 out of 533 participants at outcome assessment) compared to 26% in phase 2). 

#### Adjudication interview

The participants’ self-reported problems on the checklist were validated in a follow-up interview. In 4 cases out of 130 reports (3%; all pre-assessment, Table 2), the threshold for a follow-up interview (based on checklist data) was met, but after detailed adjudication, the reported problem did not meet the criteria for an (S)AE. Reasons were that the reported event was not new or remarkably worsened (i.e. longstanding back pain, conflicts with sibling, caregivers’ migraine, caregivers’ conflicts with own parents, caregiver’s anxiety about child’s medical problems). Very few reported AEs met the criteria for severe life-threatening problems (total of *n* = 10 SAE). Mild problems (not classified as AE, e.g. mild pain) were reported by 15% to 39% of families (ESM 3, Table 2).

**Table 2:** Frequency of reported problems with moderate-to-severe severity (checklist data)

	**Pre-assessment** *N* = 823		**After session 1** *N* = 649		**After session 3** *N* = 556		**After session 5** *N* = 537		**Follow-up assessment** *N* = 533	
	Parent	Child	Parent	Child	Parent	Child	Parent	Child	Parent	Child
Any event (*n* %)										
PLH	37 (9%)	18 (4%)	22 (6%)	19 (5%)	13 (4%)	12 (4%)	8 (3%)	6 (2%)	13 (5%)	13 (5%)
Lecture	40 (10%)	24 (6%)	18 (6%)	17 (6%)	3 (1%)	11 (4%)	5 (2%)	1 (< 1%)	17 (7%)	13 (5%)
Physical problems										
PLH	14 (4%)	3 (1%)	6 (2%)	4 (< 1%)	5 (2%)	1 (< 1%)	4 (2%)	3 (1%)	3 (1%)	1 (< 1%)
Lecture	14 (4%)	1 (< 1%)	4 (1%)	1 (< 1%)	1 (< 1%)	3 (1%)	1 (< 1%)	1 (< 1%)	5 (2%)	1 (< 1%)
Behavioural problems										
PLH	11 (3%)	3 (1%)	4 (1%)	4 (1%)	4 (1%)	1 (< 1%)	6 (2%)	2 (1%)	2 (1%)	1 (< 1%)
Lecture	9 (2%)	3 (1%)	3 (1%)	6 (2%)	1 (1%)	2 (1%)	0	0	5 (2%)	1 (< 1%)
Emotional problems										
PLH	13 (3%)	5 (1%)	9 (3%)	2 (1%)	4 (1%)	2 (1%)	8 (3%)	0	4 (2%)	2 (1%)
Lecture	19 (5%)	5 (1%)	7 (3%)	2 (1%)	2 (1%)	3 (1%)	1 (< 1%)	1 (< 1%)	4 (2%)	0
Significant problem in daily life										
PLH	24 (6%)	15 (4%)	17 (5%)	16 (4%)	8 (3%)	10 (3%)	4 (1%)	3 (1%)	9 (3%)	12 (4%)
Lecture	35 (8%)	21 (5%)	13 (5%)	11 (4%)	2 (1%)	7 (3%)	4 (1%)	0	10 (4%)	13 (5%)

Pre-assessment AE collection served as a comparison to assist in evaluating the frequency during the intervention (time frame 4 weeks). Displayed are the parents’ responses on the checklist per time point merged for each category (e.g. at least one item assessing physical problem rated with severity of 3 or 4); any event: at least one problem with severity of 3 or 4 reported across categories

#### Associations with other measures

We looked at associations between family characteristics at baseline and the occurrence of AEs during the intervention. The report of AEs during the intervention was as expected, positively associated with child mental health problems (*r* = 0.15, *p* < 0.001), as well as parent emotional problems (*r* = 0.14, *p* < 0.001) at baseline: the more parent or child mental health problems were reported at pre-intervention, the higher was the risk to report at least one AE or SAE during the intervention. In participating families with child mental health problems within one standard deviation around the CBCL mean at baseline, the prevalence of AEs during the intervention was 11% compared to 15% in the total sample. In families with child mental health problems with *t-*scores of 60 or more the percentage was higher (21%; *n* = 45 from 211). The CBCL total mean score was *M* = 58.6 (*SD* = 13.2) in the group of families with at least one (S)AE during the intervention compared to *M* = 53.9 (*SD* = 10.6) in the group without (S)AE. The amount of social support as perceived by the parent at baseline was, contrary to our expectation, not associated with AE reported during the intervention (*r* = 0.01, *p* = 0.979).

### Frequencies of AE

In total, 126 (19%) families reported at least one AE *during the intervention.* Per assessment point, the percentages of participating families with at least one AE were 10% after the first session (*n* = 65 out of 648 with AE data), 7% after the third session (*n* = 40 out of 553), and 4% after the last session (*n* = 21 out of 537). Some families (17%, *n* = 22) reported more than one AE (for details see Table 3). In the 4 weeks before the intervention and the time between post- and 1-year follow-up, we received slightly more AE reports compared to the second and third assessments during the intervention. About 9% of the sample reported at least one AE at *pre-assessment* (*n* = 77 out of 812) and at 1-year *follow-up assessment* (*n* = 50 out of 527).

**Table 3:** Characteristics of (serious) adverse events (after follow-up interview)

	**Pre-assessment** ***N* = 823**		**After session 1** ***N* = 649**		**After session 3** ***N* = 556**		**After session 5** ***N* = 537**		**Follow-up assessment** ***N* = 533**	
	**Lecture**	**PLH**	**Lecture**	**PLH**	**Lecture**	**PLH**	**Lecture**	**PLH**	**Lecture**	**PLH**
Total *N*	408	415	285	364	255	301	229	308	247	286
AE data available	401	411	284	364	253	301	229	308	244	283
Follow-up call required	42 (10%)^a^	45 (11%)	32 (12%)^a^	35 (10%)	17 (7%)^a^	24 (8%)	7 (3%)^a^	15 (5%)	28 (12%)	27 (10%)
Results of follow-up interview										
No AE	3 (< 1%)	1 (< 1%)	1 (< 1%)	0	0	0	0	0	0	0
AE	35 (9%)	42 (10%)	30 (11%)	35 (10%)	16 (5%)	24 (8%)	6 (3%)	15 (5%)	27 (11%)	23 (8%)
SAE	3 (< 1%)	2 (< 1%)	0	0	0	0	0	0	1 (< 1%)	4 (1%)
Happened to										
Caregiver	31 (8%)	35 (9%)	14 (5%)	22 (6%)	4 (2%)	14 (4%)	4 (2%)	8 (3%)	14 (6%)	14 (5%)
Child	10 (2%)	10 (2%)	16 (6%)	13 (4%)	12 (5%)	9 (3%)	2 (1%)	7 (2%)	13 (5%)	11 (4%)
Expected	21 (5%)	21 (5%)	13 (5%)	11 (3%)	4 (2%)	7 (2%)	4 (2%)	5 (2%)	9 (4%)	8 (3%)
Definitely/probably/possibly related to study	0	0	0	0	0	0	0	0	0	0
Unrelated to study^b^	38 (100%)	44 (100%)	30 (100%)	35 (100%)	16 (100%)	24 (100%)	6 (100%)	15 (100%)	28 (100%)	27 (100%)
COVID-19 related	7 (2%)	3 (1%)	4 (2%)	9 (3%)	1 (< 1%)	4 (1%)	0	2 (1%)	5 (19%)	4 (17%)
*N* of cases with more than one AE										
2 AE	8 (2%)	13 (3%)	2 (1%)	5 (1%)	3 (1%)	4 (1%)	0	3 (1%)	6 (3%)	6 (3%)
3 AE	4 (1%)	7 (2%)	1 (< 1%)	2 (1%)	3 (1%)	2 (1%)	0	0	2 (1%)	1 (< 1%)

Note*.* Displayed is the number of families that reported at least one AE per assessment point in both conditions. Percentages relate to the cases per randomised group with available AE data at each assessment point

^a^*n* = 1 case with required follow-up call according to checklist data, but follow-up call form is missing

^b^Percentages relate to the cases with at least one (S)AE per randomised group at each assessment point

The frequency of AE reports was similar in both groups during and after the intervention (Table 3). We received a total of 74 AE reports in the PLH condition *during the intervention*, compared to 52 in the lecture group (see Table 3). There were 23 AE reports in the PLH-YC group (8%) *after the intervention*, compared to 27 in the lecture group (11%).

The most frequent categories, with moderate-to-severe severity across the three assessment points during the intervention, were parents’ emotional problems (*n* = 31), parents’ physical problems (pain *n* = 20), child behavioural problems (*n* = 15), and child emotional problems (*n* = 10). Unplanned hospitalisation, accidents, and injuries were rarely reported (see Table 1 ESM 3 and Table 2). The frequencies of (expected) problems (e.g. short-term increase of child behaviour problems, emotional problems) were numerically not higher in the intervention group compared to the control group (Table 2). For completeness, the frequency of mild levels of distress experienced during the trial (not classified as AE) are displayed in the ESM 3 (Table 2: Checklist results with severity of 1 or 2 (mild problems).

### Frequencies of SAE

In total, 1% of participating families (*n* = 10) reported an SAE during the trial. Half were reported before the intervention (pre-assessment: *n* = 5), and half afterwards (follow-up assessment: *n* = 5). No SAE occurred during the intervention. The risk for SAE was similar for both groups (intervention and control) during and after the intervention (see Table 3).

At *pre-assessment*, SAEs included in the lecture group: (1) child hospitalised due to bronchitis, (2) parent hospitalised due to birth complications, and (3) child’s surgery due to genital problem since birth. In the PLH-YC group, SAEs were (1) parent hospitalised with a broken leg and (2) child hospitalised with a cold. At *follow-up assessment*, one SAE was reported in the lecture group: child hospitalised due to leg problems. In the PLH-YC group, SAEs included (1) unplanned hospitalisation of child due to bacterial infection, (2) child hospitalised due to planned surgery, (3) child hospitalised with a broken leg, and (4) caregiver’s emotional problems due to death of close relatives (Table 3).

### Adverse reactions/negative effects

We did not find any evidence for the intervention or study procedures generating the detected problems (no causal relationship). All AEs and SAEs were determined as unrelated to the study based on adjudication of implementation site PI and consultation with DSMB. In addition to the structured assessment results, the local research teams reported some protest from families in the control condition: some of the parents were unhappy because they had only received one lecture but needed to complete several hours of assessment. Assessment burden per family included the three outcome assessments (each lasting about an hour), along with three additional brief interviews during the intervention. The research staff did not register any specific dissatisfaction of parents related to the AE assessment (duration of about 5 min).

## Summary and concluding discussion

The aim of this paper was to develop a systematic AE assessment tool for parenting interventions over three consecutive studies and test the optimised AE assessment procedure in the final multisite RCT.

Quality and quantity of the AE data improved over the three consecutive studies. Whilst the AE response rates in phases 1 and 2 were insufficient, especially during the intervention, we received AE data from all participants in phase 3. To examine our hypotheses that more AEs may occur in families with more (severe) mental health problems, we analysed the associations between baseline child and parent mental health problems and AE reports during the intervention as a potential indicator for the validity of the AE assessment procedures. AE reports during the intervention were significantly associated with baseline parent and child mental health problems (small effect sizes) but not with perceived social support.

Overall, we found very low levels of AEs with our active assessment method. During the intervention, about 10% of families reported at least one new problem or a significant worsening of a pre-existing AE; no family experienced a SAE. Most importantly, none of the reported (S)AEs was an adverse reaction perceived to have a causal relationship to the intervention or study. With regard to conditions, the frequencies were similar in the intervention group (five sessions of the PLH-YC parenting programme) and the control group (one lecture on parenting). The most frequently reported categories of problems in our sample were emotional distress and physical problems (e.g. pain) in parents. There was a (numerical) decrease of reported SAEs and AEs during the intervention compared to baseline assessment (not examined statistically because of small sample size). The overall frequencies were small (1 to 5% depending on assessment point) and these problems are common in the general population. Also, some cases that were correctly classified as SAEs according to the SAE criteria (besides others: hospital admission with more than one night) were of moderate severity (e.g. child hospitalised with a cold). With these experiences in mind, we would now recommend being more specific in parenting intervention research regarding hospitalisation as a clear indication for a SAE based on the reason for hospitalisation: Whilst unplanned emergency visits (which are typical for child injuries that could be but are mostly not a result from family violence) should be part of SAE definition, as well as all other reasons that may be a consequence from violence (broken bones, burns, unclear reasons), others may be taken out, such as hospitalisations due to infectious diseases. Moreover, contextual factors of health service systems need to be considered for the development of (S)AE criteria: feedback from the local research teams suggested that it was known practice in some research areas to visit the hospital instead of scheduling an appointment with a doctor in case of mild-to-moderate medical problems (such as moderate fever, cold) often due to health insurance reasons and local infrastructure constraints. Moreover, the long waiting times to secure appointments with specialists and the primary health system being overstretched with COVID infections may have prompted families to seek immediate hospital care. We also registered some planned hospitalisations (e.g. planned surgery) that were clearly unrelated to the study participation. Based on these experiences, it might be reasonable to contextualise the SAE criteria in future studies to the specific health service system and differentiate the reason for hospitalisation to disentangle SAE from AE. Finally, the detected problems may rather reflect the everyday life of families experiencing difficulties with their children rather than adverse effects caused by the study or intervention. This is supported by the finding that family adversities (i.e. parent or child mental health problems) were associated with AE occurrence during the intervention: Families with more parent or child mental health problems were more likely to report at least one (S)AE during the intervention.

The applied assessment mode (conducted by data assessors in-person or over the phone) may have affected the parents’ willingness to report AEs. Because we experienced, during the earlier study phases, that the group leaders did not have the time to collect data on AE during the parent programme sessions, we left this task to the data assessors. The parents knew their data assessor from prior assessments (the same data assessor assessed each participant at different time points, if feasible). However, they may have reported more problems if the group leaders (whom they knew and trusted) would have asked the AE questions. Thus, the AE assessment by data assessors may have resulted in an underreporting of AE (particularly on emotional and behavioural problems). However, it is also possible that parents would have reported less problems to the group leaders (compared to a more anonymous data assessor), not wanting to admit problems especially towards the completion of the programme.

At pre-assessment, four cases scored positively on the checklist but did not meet criteria for an AE or SAE in the adjudication interview. These false positive reports did not occur during the later assessment points (apart from one case at the first assessment during the intervention). Based on feedback from the research coordinators, this could possibly reflect a learning curve for both assessors and parents. After the initial experiences with the checklist at pre-assessment, the definition of an (S)AE was clearer. When parents reported a problem, the data assessors asked directly whether the problem was really new or remarkably worsened. This finding, as well as our experiences in the development of the AE procedures in the RISE project (phases 1 and 2), underlines the importance of training the data assessors in AE assessment, especially when they have limited knowledge in (clinical) psychology and might not be familiar with AE concepts or mental health problems. However, we cannot exclude the possibility that the decrease of (S)AEs during the intervention was (at least partly) due to selective dropout (57% of families in the lecture group attended all assessments compared to 64% in the PLH-YC group) and that families with more adversities and a higher risk of AEs did not participate in the later assessment points. In line with this, there was feedback from some parents in the control condition that they were feeling that they received less help. As a consequence, some parents may have declined to participate in the assessments; others might have been less open (and reported less on personal problems). This might reflect an unwanted negative effect of the study design (randomised group allocation).

Even though our active approach tended towards over-reporting AEs (e.g. or detecting problems that were minor and did not meet the threshold for an AE), we applied it to ensure that no potential AEs were missed with the aim to increase systematic knowledge on AEs in parenting interventions (what categories, frequency, and severity of problems can be expected during a trial). Our study thus adds an important finding to the existing literature [15, 16, 30] on AEs in parenting interventions. With a very sensitive approach of active AE assessment, we found similar rates of SAEs compared to other studies with none of these problems being caused by the intervention (risk for SAEs in our trial at the lower bound of studies). This underlines the conclusions from a recent large WHO Guideline review [9] that, based on a range of methods, there were no harms detected across multiple parenting intervention trials. However, the sensitive AE assessment was a time burden for data assessors and families (approximately 5–15 min per assessment point). Thus, once a comprehensive database on AEs in parenting interventions is established, and if all studies across samples, research groups, and intervention types consistently demonstrate that studied parenting interventions did not cause any harm, it might be possible to reduce the (time) burden for AE assessment (at least in some studies) as benefits of comprehensively assessing these events might not exceed its costs. One option could be to set a higher threshold (e.g. only follow-up on reported severe problems) and to shorten the active assessment (e.g. go back to the one or two open questions) [28] to reduce the time burden for AE assessment in population, settings, and interventions with lower risk profiles (e.g. less family adversities). This increases the risk of missing AEs again but on a background of our finding (amongst others) suggesting that the implemented parenting programme appeared safe in this setting and with this population, this might be a viable option.

## Strengths and limitations

In the follow-up interview, we evaluated the parent-reported problems (to check whether or not the criteria for an AE were met). However, we did not cross-check the parents’ reports on the AE checklist (e.g. double-check with group leaders if any problems were reported during the sessions) because the data assessors needed to be blind to the group allocation. It cannot be ruled out that parents underreported AE related to their own emotional and behavioural problems and emotional problems related to the children. Also, validation work for the AE tool (e.g. associations with other measures) was limited due to the low base rate of (S)AEs.

Missingness occurred due to dropout of participants. Missing AE checklist or follow-up interview data occurred in very few cases (< 1%). However, missing data in AE assessment is especially important because one case could have a large effect on the study (e.g. SAE related to the intervention). Thus, we applied the following steps to minimise missingness. First, data assessors and research coordinators were trained in the AE checklist by using tablet technology. The tablet software sent an automatic reminder if the threshold for a follow-up interview was met—based on AE checklist data. We tested the AE assessment in the prior project phases and optimised the procedures based on the experiences obtained to minimise missingness. Finally, the focus of the overall project was on the implementation (following RE-AIM) [35] at a large scale rather than on high internal validity.

Due to the COVID pandemic restrictions, we needed to switch from in-person interviews to phone interviews. For remote assessments, it was easier to schedule the interviews with parents because they did not need to travel and organise child care for an in-person interview outside their homes. On the downside, the local research teams reported that some parents were not able to fully concentrate on the assessments because they were actively involved in child care at the same time. To avoid any potential risk of bias, we recommend the use of in-person assessments where possible.

Important strengths of the study were the iterative development process of the AE procedures over three project phases, the large sample across three countries, and the systematic two-stepped AE assessment method (checklist and follow-up interview) to minimise the risk of missing potential negative reactions. These strengths enhance the reliability and comprehensiveness of the study’s findings.

## Directions for future studies

Regarding the definition and reporting of AEs, we recommend the application of recent guidelines, such as CONSORT [1, 2, 11], to facilitate the comparison of AE results across studies. Criteria for AE, SAE, and adverse reactions can be adapted for the specific field as needed.

Based on our experience, we encourage researchers that aim to comprehensively assess AEs to apply our two-stepped approach. This sensitive procedure minimises the risk of missing any important adverse effects. The self-report checklist as well as the adjudication form should be tailored to the specific field (e.g. what are expected AE and adverse reactions). Moreover, based on our experiences in phases 1 and 2, we strongly recommend testing the AE assessment tool beforehand. This enables optimisation of the procedures to fit the specific context (e.g. sample and intervention characteristics) and thereby enhances the quality and completeness of AE data.

When planning the AE assessment methods, researchers might want to carefully weigh up the benefits and costs of a comprehensive time and cost-intense active assessment procedure. In psychological intervention studies with participants reporting clinical levels of mental health problems, it seems crucial to comprehensively assess AEs using an active assessment strategy [1, 2]. However, in prevention studies with healthy individuals, the risk for AEs seems smaller—especially for AEs with likely causal relationship to study participation [9]. So, the benefits of a comprehensive active AE assessment might not outweigh the costs (financial costs for research, time costs for participants). A passive AE assessment or a brief active assessment (e.g. one or two open questions) might be sufficient.

The picture for selective and indicated samples with elevated levels of problems is less clear. It is likely that the risk of AE increases with higher levels of child mental health problems. Based on our results, a reasonable cutoff to switch from passive to active AE assessment might be when a meaningful percentage of the sample scores one standard deviation above the mean (e.g. child mental health problems: CBCL *t-*scores of 60 or higher). Instead of using the active AE assessment for all participants, researchers could also think about applying this more comprehensive assessment for higher risk families only (e.g. above the cutoff)—especially when resources are limited. However, other study and sample characteristics (e.g. new intervention, sample characteristics) should be also considered when planning AE assessment methods. Finally, the suggested cutoff would need to be validated in other studies, samples, and intervention types. This seems crucial as parenting interventions can be built on varied techniques, approaches, and formats that may relate to different potential AEs (e.g. higher risks of marital conflicts about parenting strategies in programmes where these are taught, stronger feelings of guilt when gaining insight into own parenting behaviour that qualifies for child maltreatment, or frustration with the child for lack of responses to new parenting strategies). Finally, the population and setting in which the interventions are used seem highly relevant for estimating safety risks. In samples with more adversities such as a low socio-economic status or high prevalence of mental health and medical problems, the risk of AEs might be higher. We recommend the collection of data on the involvement of child protection services, child being removed from the household, school disciplinary actions, domestic abuse, and police involvement if possible in trials including youth with elevated behaviour problems. For trials focusing on other mental health problems (e.g. child anxiety), these specific items may be less relevant. Whilst information based on records might be more objective, initially using only parent report seems less resource intense. Also, further details about the event can be gathered from parents. Finally, local research teams reported that contextual factors (e.g. distrust of authorities, norms of privacy) might defer participation if families needed to agree to access to external records.

## Conclusions

This paper is the first of its kind to rigorously implement a comprehensive AE assessment method of a parenting programme using a systematic procedure that was developed and tested in three sequential studies with more than 1500 families in three countries and provides much-needed evidence for interventions conducted and evaluated in low- and middle-income countries. It contributes towards a comprehensive standardised AE assessment in parenting programme research and increases knowledge about safety in this field. Based on the given literature, we have clear evidence of the beneficial effects of parenting interventions (i.e. reduction of child behaviour problems and dysfunctional parenting behaviour) [9], whilst finding no harms.

Increasing systematic knowledge on AEs in parenting interventions by using comprehensive AE assessments and reports as an integral part of future parenting intervention studies to ensure their safety. Based on our experiences, we recommend piloting the AE assessment methods and applying an active rather than passive assessment method—especially for high-risk samples [6]. To overcome the challenge of varying AE definitions, assessment methods, and reporting practices across studies, we welcome other researchers to consider applying our checklist and follow-up interview (with adaptations to the specific context). This collaborative effort would facilitate the comparison of (S)AE across different intervention types, samples, and conditions. Ultimately, in the long-term, this will allow informed treatment decisions as well as recommendations based on a comprehensive assessment on potential benefits and harms in parenting interventions.

### Supplementary Information

Supplementary Material 1

Supplementary Material 2

Supplementary Material 3

Supplementary Material 4

## Data Availability

The AE materials (AE checklist and follow-up form) are openly accessible in ESM 1 and 2 of this paper. Some of the study resources (e.g. PLH-YC work book, lecture slides) are available on the RISE website (https://rise-plh.eu/). Following the EU guidelines, all RISE papers were and will be submitted to open-access peer-reviewed journals. According to the FAIR (findable, accessible, interoperable, reusable) principles, some of the anonymized RISE data sets will be available for others via an open-access repository. For questions regarding access to RISE data, please contact HMF (heather.foran@aau.at) who is responsible for overall data management for the RISE project.

## Abbreviations

AE: Adverse event
ADHD: Attention-deficit hyperactivity disorder
CABI: Child and adolescent behaviour inventory
CBCL: Child behaviour checklist
CD: Conduct disorder
DASS: Depression Anxiety Stress Scales
DSMB: Data safety and monitoring board
FAIR: Findable accessible interoperable reusable
IRB: Institutional review board
MOS: Medical Outcomes Study
ODD: Oppositional defiant disorder
PI: Principal investigator
RCT: Randomised controlled trial
RISE: Prevention of child mental health problems in Eastern Europe
SAE: Serious adverse events
