# Supplementary figures and images for: Adverse event assessment in a parenting programme: experiences from a multisite randomised controlled trial
Source: Trials. 2024 Aug 17;25:547. doi: 10.1186/s13063-024-08357-6 (PMC11330034; doi:10.1186/s13063-024-08357-6)

*ESM 1*

*Adverse event record Phase 1*


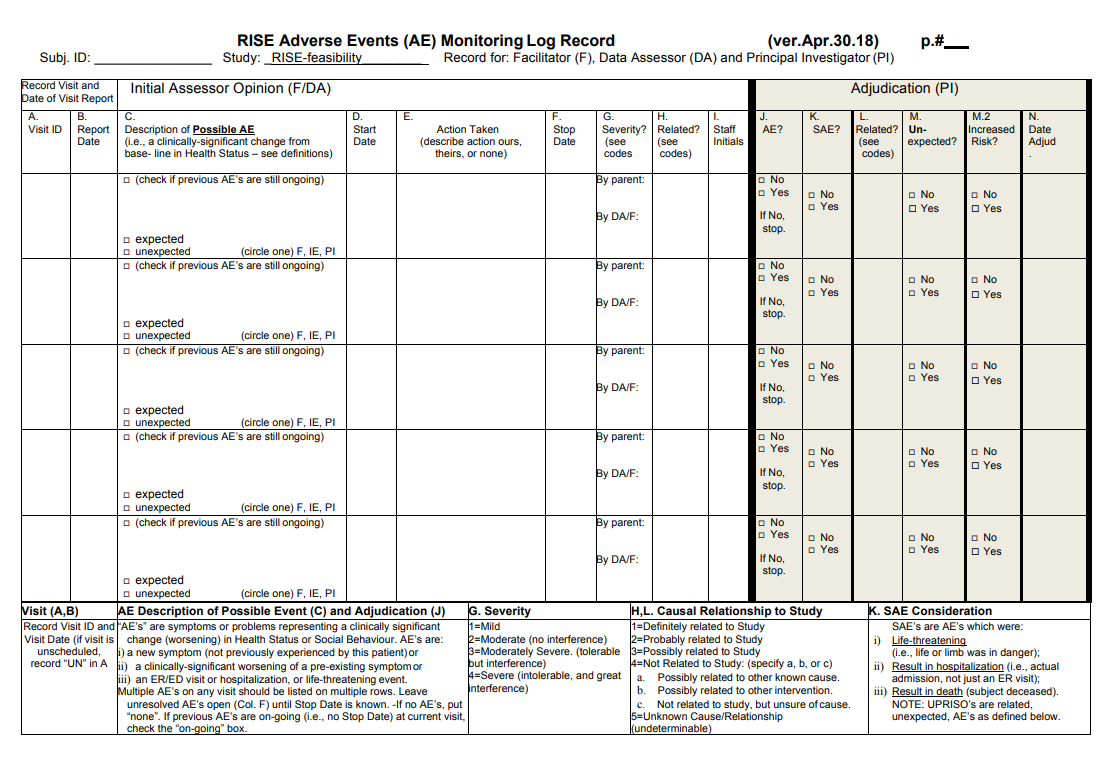


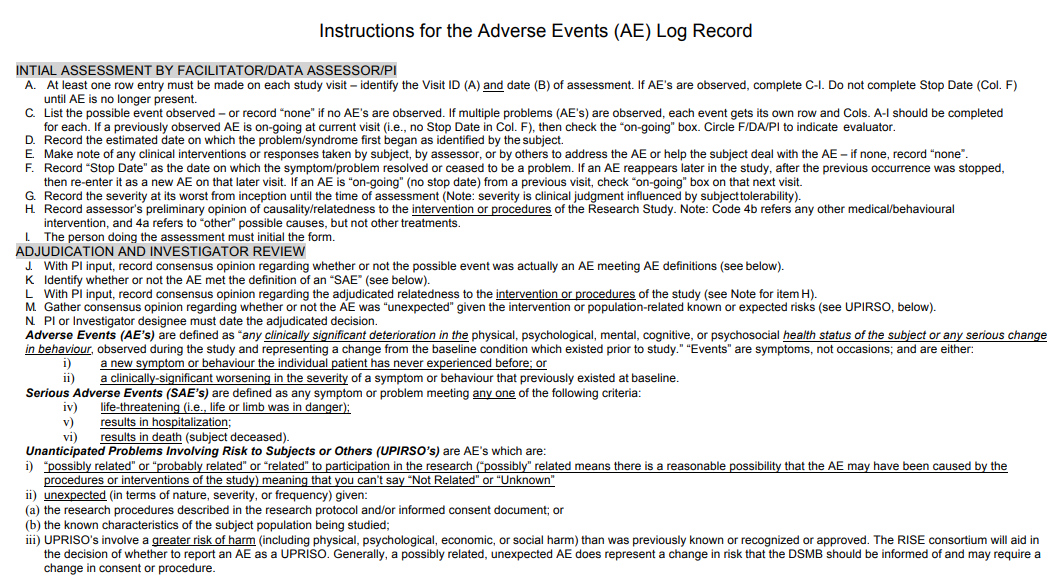

Supplement: Supplementary file 1 — Supplementary Material 1 [file 13063_2024_8357_MOESM1_ESM.docx]
