# Supplementary material for: Adverse event assessment in a parenting programme: experiences from a multisite randomised controlled trial
Source: Trials. 2024 Aug 17;25:547. doi: 10.1186/s13063-024-08357-6 (PMC11330034; doi:10.1186/s13063-024-08357-6)
Supplement: Supplementary file 2 — Supplementary Material 2 [file 13063_2024_8357_MOESM2_ESM.docx]

*ESM 2*

*AE Checklist in RISE (example from pre-assessment Phase 3)*

**What happened to my family in the last 4 weeks?**

**Family ID: __________________ Date: __________________**

| Check **“No”** if nothing of the below has happened to you or your child in the last 4 weeks.  Check **“Yes”** if one of these issues **newly occurred or worsened** in the last 4 weeks.  If **“Yes”**, circle the number showing how severely it affected you or your child (1 = mild, 4 = severe).  **In the last 4 weeks, did you or your partner or your child suffer newly occurring problems or those that got worse, such as…** | | | | |
| --- | --- | --- | --- | --- |
|  | **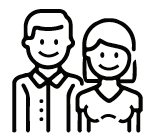Did not happen to parent** | **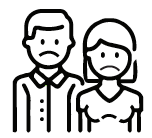Happened to parent** | **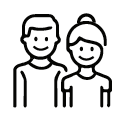Did not happen to child** | **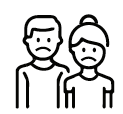Happened to child** |
| Physical/Medical Problems | | | | |
| 1. 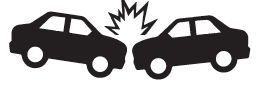**…Accident** | **No** | **Yes**  **1 – 2 – 3 – 4** | **No** | **Yes**  **1 – 2 – 3 – 4** |
| 1. 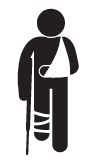**…Injury** | **No** | **Yes**  **1 – 2 – 3 – 4** | **No** | **Yes**  **1 – 2 – 3 – 4** |
| 1. 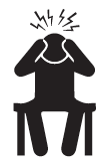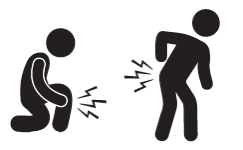**…Pain (e.g., headaches, back pain)** | **No** | **Yes**  **1 – 2 – 3 – 4** | **No** | **Yes**  **1 – 2 – 3 – 4** |

|  | **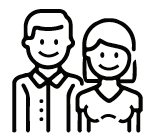Did not happen to parent** | **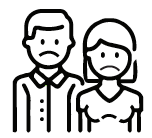Happened to parent** | **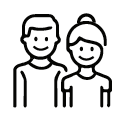Did not happen to child** | **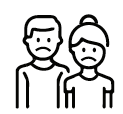Happened to child** |
| --- | --- | --- | --- | --- |
| Behavioral Problems | | | | |
| 1. …Aggressive / violent behaviors   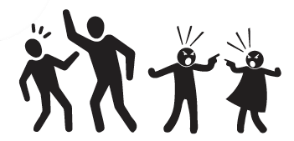 | **No** | **Yes**  **1 – 2 – 3 – 4** | **No** | **Yes**  **1 – 2 – 3 – 4** |
| 1. **…Sleep disturbances**   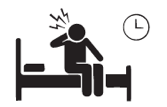 | **No** | **Yes**  **1 – 2 – 3 – 4** | **No** | **Yes**  **1 – 2 – 3 – 4** |
| 1. **…Alcohol / drug / medication use**   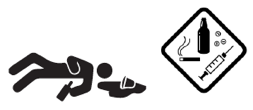 | **No** | **Yes**  **1 – 2 – 3 – 4** | **No** | **Yes**  **1 – 2 – 3 – 4** |
| **Emotional Problems** | | | | |
| 1. **…Emotionally distressed (e.g., feeling anxious, nervous, sad, depressed, angry)**   **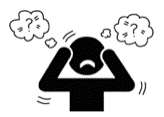**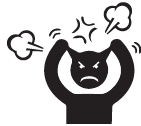  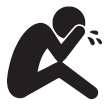 | **No** | **Yes**  **1 – 2 – 3 – 4** | **No** | **Yes**  **1 – 2 – 3 – 4** |

|  | **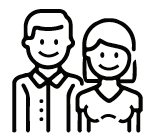Did not happen to parent** | **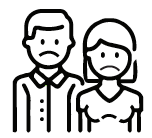Happened to parent** | **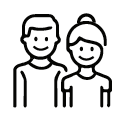Did not happen to child** | **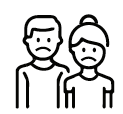Happened to child** |
| --- | --- | --- | --- | --- |
| Significant problems in daily life | | | | |
| 1. **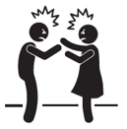…Difficulties with personal relationships (e.g., conflict with a friend, divorce, parent-child conflict) or daily responsibilities (e.g. not go to nursery or school)** | **No** | **Yes** | **No** | **Yes** |
| 1. 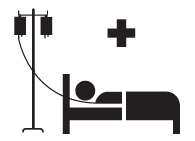**…Unplanned hospitalization** | **No** | **Yes** | **No** | **Yes** |
| 1. 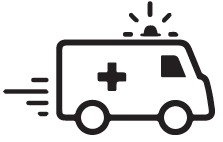**…Emergency room visits** | **No** | **Yes** | **No** | **Yes** |
| 1. 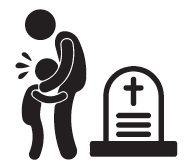**…Death of a loved one­** | **No** | **Yes** | **No** | **Yes** |
| 1. **…Any other problems (incl. medical, emotional, behavioral)? Please specify:**   ----------------------------- | **No** | **Yes** | **No** | **Yes** |

**ESM 2: AE Follow-up Interview (completed by research staff)**

| **Adjudication** (please complete in English) | | | | | | | | | | | | | | | |
| --- | --- | --- | --- | --- | --- | --- | --- | --- | --- | --- | --- | --- | --- | --- | --- |
| Staff initials: ________________ | | | | | | Date: ________________ | | | | | | | | | |
| ***1st Adverse event*** | | | | | | | | | | | | | | | |
| O happened to *child*  O happened to *caregiver* | | | | | **Was the event expected?** O Expected  O Unexpected | | | | | | | | | | |
| **O Adverse event** | | | | | | | | **O Serious adverse event** | | | | | | | |
| - *a new symptom (not previously experienced) or* - *a clinically-significant worsening of a pre-existing symptom or* - *an Emergency Room visit* | | | | | | | | - *Life-threatening (i.e., life or limb was in danger)* - *Result in hospitalization (i.e., actual admission at least one night, not just an ER visit)* - *Result in death (participant died)* | | | | | | | |
| **Detailed description event/action taken:** | | | | | | | | | | | | | | | |
| **How severe was the event (in your opinion)?** | | | | | | | | | | | | | | | |
| **1** | | | **2** | | | | | | | **3** | | | **4** | | |
| Mild | | | Moderate  (no interference) | | | | | | | Moderately Severe  (tolerable but interference) | | | Severe (intolerable, and great interference) | | |
| **Is there a causal relationship to the study?** | | | | | | | | | | | | | | | |
| **1** | | **2** | | | | | **3** | | | | **4** | | | **5** | |
| Not Related to  Study  **Specify:**  a) Possibly related to other known cause  b) Possibly related to other intervention  c) Not related to study, but unsure of cause | | Unknown Cause/  Relationship  (undeterminable) | | | | | Possibly related to Study | | | | Probably related to Study | | | Definitely related to Study | |
| **What is the outcome?** | | | | | | | | | | | | | | | |
| **1**  Recovery  (no remaining symptoms at follow-up call) | **2**  Recovery with some remaining symptoms (acute symptoms disappeared but subclinical problems remain at follow-up call) | | | **3**  Improvement (symptoms less severe at follow-up call) | | | | | **4**  No recovery  (symptoms did not improve at follow-up call) | | | **5**  Death | | | **6**  Unknown  (also: caregiver report not plausible) |

*If any adverse event was reported,* ***please report this to your country PI*** *(xxxx) within 24 hours* ***who needs to*** ***inform the Coordinator*** *(xxx) within 72 hours (in case of a SAE, the coordinator need to report to DSMB)*

***When*** *local law of child maltreatment is met, report to local child protection service.*

| ***2nd Adverse event*** | | | | | | | | | | | | | | |  |
| --- | --- | --- | --- | --- | --- | --- | --- | --- | --- | --- | --- | --- | --- | --- | --- |
| O happened to *child* | | | | | **Was the event expected?** O Expected | | | | | | | | | |  |
| O happened to *caregiver* | | | | | O Unexpected | | | | | | | | | |  |
| **O Adverse event** | | | | | | | **O Serious adverse event** | | | | | | | |  |
| - *a new symptom (not previously experienced) or* - *a clinically-significant worsening of a pre-existing symptom or* - *an Emergency Room visit* | | | | | | | - *Life-threatening (i.e., life or limb was in danger)* - *Result in hospitalization (i.e., actual admission at least one night, not just an ER visit)*   *Result in death (participant died)* | | | | | | | |  |
| **Detailed description event/action taken:** | | | | | | | | | | | | | | |  |
| **How severe was the event (in your opinion)?** | | | | | | | | | | | | | | |  |
| **1** | | | **2** | | | | | | **3** | | | **4** | | |  |
| Mild | | | Moderate  (no interference) | | | | | | Moderately Severe  (tolerable but interference) | | | Severe (intolerable, and great interference) | | |  |
| **Is there a causal relationship to the study?** | | | | | | | | | | | | | | |  |
| **1** | | **2** | | | | **3** | | | | **4** | | | **5** | |  |
| Not Related to  Study  **Specify:**  a) Possibly related to other known cause  b) Possibly related to other intervention  c) Not related to study, but unsure of cause | | Unknown Cause/  Relationship  (undeterminable) | | | | Possibly related to Study | | | | Probably related to Study | | | Definitely related to Study | |  |
| **What is the outcome?** | | | | | | | | | | | | | | | |
| **1**  Recovery  (no remaining symptoms at follow-up call) | **2**  Recovery with some remaining symptoms (acute symptoms disappeared but subclinical problems remain at follow-up call) | | | **3**  Improvement (symptoms less severe at follow-up call) | | | | **4**  No recovery  (symptoms did not improve at follow-up call) | | | **5**  Death | | | **6**  Unknown  (also: caregiver report not plausible) | |

*If any adverse event was reported,* ***please report this to your country PI*** *(xxxx) within 24 hours* ***who needs to*** ***inform the Coordinator*** *(xxx) within 72 hours (in case of a SAE, the coordinator need to report to DSMB)*

***When*** *local law of child maltreatment is met, report to local child protection service*
