# Supplementary material for: Adverse event assessment in a parenting programme: experiences from a multisite randomised controlled trial
Source: Trials. 2024 Aug 17;25:547. doi: 10.1186/s13063-024-08357-6 (PMC11330034; doi:10.1186/s13063-024-08357-6)
Supplement: Supplementary file 3 — Supplementary Material 3 [file 13063_2024_8357_MOESM3_ESM.docx]

*ESM 3*

*Table 1*

*Parent-reported Frequency of Problems with Moderate to Severe Severity (Checklist-data): Per Item across Conditions*

|  | **Pre-assessment**  *N* = 823 | | **After session 1**  *N* = 664 | | **After session 3**  *N* = 568 | | **After session 5**  *N* = 547 | | **Follow-up assessment**  *N* = 543 | |
| --- | --- | --- | --- | --- | --- | --- | --- | --- | --- | --- |
| Happened to | Parent | Child | Parent | Child | Parent | Child | Parent | Child | Parent | Child |
| **Physical problems** | | | | | | | | | | |
| Accident | 0 | 0 | 1 (<1%) | 1 (<1%) | 0 | 0 | 0 | 1(<1%) | 0 | 0 |
| Injury | 2 (<1%) | 2 (<1%) | 1 (<1%) | 0 | 0 | 2 (<1%) | 1 (<1%) | 2 (<1%) | 1 (<1%) | 1 (<1%) |
| Pain | 26 (3%) | 2 (<1%) | 9 (1%) | 4 (1%) | 6 (1%) | 2 (<1%) | 5 (1%) | 4 (1%) | 7 (2%) | 0 |
| **Behavioral problems** | | | | | | | | | | |
| Aggressive | 4 (1%) | 4 (<1%) | 3 (1%) | 10 (2%) | 0 | 1 (<1%) | 2 (<1%) | 1 (<1%) | 0 | 1 (<1%) |
| Sleep problems | 15 (2%) | 3 (<1%) | 4 (1%) | 0 | 5 (1%) | 1 (<1%) | 3 (1%) | 1 (<1%) | 6 (2%) | 1 (<1%) |
| Substance use | 3 (<1%) | 0 | 2 (<1%) | 1 (<1%) | 0 | 1 (<1%) | 1 (<1%) | 0 | 1 (<1%) | 1 (<1%) |
| **Emotional problems** | | | | | | | | | | |
|  | 32 (4%) | 10 (1%) | 16 (3%) | 4 (1%) | 6 (1%) | 5 (1%) | 9 (2%) | 1 (<1%) | 8 (2%) | 2 (1%) |
| **Significant problems in daily life (Yes)** | | | | | | | | | | |
| Conflicts | 24 (3%) | 12 (1%) | 11 (2%) | 9 (1%) | 5 (1%) | 3 (1%) | 3 (<1%) | 0 | 6 (2%) | 2 (1%) |
| Hospitalization | 4 (<1%) | 2 (<1%) | 0 | 0 | 0 | 0 | 0 | 1 (<1%) | 0 | 3 (1%) |
| Emergency room | 10 (1%) | 8 (1%) | 3 (<1%) | 6 (1%) | 2 (<1%) | 7 (1%) | 2 (<1%) | 1 (<1%) | 5 (1%) | 12 (3%) |
| Death loved one | 12 (2%) | 8 (1%) | 10 (2%) | 5 (1%) | 1 (<1%) | 1 (<1%) | 2 (<1%) | 0 | 5 (1%) | 2 (1%) |
| Other problem | 13 (2%) | 10 (2%) | 14 (2%) | 9 (1%) | 5 (1%) | 7 (1%) | 2 (<1%) | 2 (<1%) | 4 (1%) | 8 (2%) |

**Note.** Displayed percentages are the % of parents that reported this problem on the checklist per assessment point. Severity rating of 3 or 4 on the AE checklist (for physical, behavioral and emotional problems). Significant problem in daily life: response options: happened yes/no (no severity rating).

*ESM 3*

*Table 2*

*Frequencies of Mild Problems Reported by Parents (not Classified as AE, Checklist-data)*

|  | **Pre-assessments**  *N* = 836 | | **After session 1**  *N* = 664 | | **After session 3**  *N* = 568 | | **After session 5**  *N* = 547 | | **Follow-up assessment**  N = 543 | |
| --- | --- | --- | --- | --- | --- | --- | --- | --- | --- | --- |
|  | Parent | Child | Parent | Child | Parent | Child | Parent | Child | Parent | Child |
| **Any Event,** *N* % |  |  |  |  |  |  |  |  |  |  |
| PLH | 162 (39%) | 146 (36%) | 117 (32%) | 123 (34%) | 93 (31%) | 84 (28%) | 47 (15%) | 47 (15%) | 76 (27%) | 68 (24%) |
| Lecture | 152 (38%) | 125 (31%) | 76 (27%) | 73 (26%) | 56 (22%) | 49 (19%) | 36 (16%) | 45 (20%) | 77 (32%) | 61 (25%) |
| By category |  |  |  |  |  |  |  |  |  |  |
| **Physical problems** | |  |  |  |  |  |  |  |  |  |
| PLH | 108 (26%) | 36 (9%) | 72 (20%) | 36 (10%) | 52 (17%) | 25 (8%) | 20 (7%) | 16 (5%) | 52 (18%) | 28 (10%) |
| Lecture | 109 (27%) | 28 (7%) | 40 (14%) | 22 (8%) | 28 (11%) | 17 (7%) | 18 (8%) | 27 (12%) | 49 (20%) | 25 (10%) |
| **Behavioral problems** | |  |  |  |  |  |  |  |  |  |
| PLH | 56 (14%) | 69 (17%) | 52 (14%) | 67 (18%) | 27 (9%) | 38 (13%) | 16 (6%) | 17 (6%) | 14 (5%) | 22 (8%) |
| Lecture | 55 (14%) | 62 (15%) | 36 (13%) | 33 (12%) | 17 (7%) | 23 (9%) | 17 (7%) | 11 (5%) | 30 (12%) | 19 (8%) |
| **Emotional problems** | |  |  |  |  |  |  |  |  |  |
| PLH | 87 (21%) | 44 (11%) | 70 (19%) | 38 (10%) | 52 (17%) | 28 (9%) | 23 (7%) | 14 (5%) | 35 (12%) | 14 (5%) |
| Lecture | 82 (20%) | 44 (11%) | 46 (16%) | 35 (12%) | 30 (12%) | 22 (9%) | 17 (7%) | 11 (5%) | 38 (16%) | 18 (5%) |

**Note.** Displayed are the parents’ responses on the checklist per time point and condition; merged for each category (e.g., at least one physical problem with severity of 1 or 2 reported); Any event: at least one problem with severity of 1 or 2 reported across categories. These events were not classified as AE (see AE definitions).
